# Transcriptionally Informed Nucleosome Profiling of Circulating Cell-Free DNA Predicts Breast Cancer Recurrence

**DOI:** 10.1158/2767-9764.CRC-26-0263

**Published:** 2026-06-15

**Authors:** Sugiko Watanabe, Kan Etoh, Jun Mitsui, Yuta Suzuki, Yutaka Yamamoto, Mitsuyoshi Nakao

**Affiliations:** 1 Department of Medical Cell Biology, Institute of Molecular Embryology and Genetics, Kumamoto University, Kumamoto, Japan.; 2 Department of Molecular Cell Biology, Graduate School of Medical Sciences, Kyushu University, Fukuoka, Japan.; 3 Department of Molecular Neurology, Graduate School of Medicine, The University of Tokyo, Tokyo, Japan.; 4 Department of Computer Science, Graduate School of Information Science and Technology, The University of Tokyo, Tokyo, Japan.; 5 Department of Breast and Endocrine Surgery, Kumamoto University Hospital, Kumamoto, Japan.

## Abstract

Cell-free DNA (cfDNA) offers a minimally invasive approach to capture genomic and epigenetic dynamics during cancer progression. We performed targeted sequencing of 26 gene loci transcriptionally regulated during the acquisition of therapy resistance in breast cancer and analyzed blood-derived cfDNA from 150 breast cancer samples (105 primary and 45 recurrent). Recurrent samples exhibited increased genomic variant counts in both coding and noncoding regions, accompanied by shorter cfDNA fragment lengths. Furthermore, cfDNA fragmentation profiles were variable in recurrent samples, with frequently amplified loci such as *ERBB2* and concurrent reductions at loci, including *RERE* and *SYNPO2*. Notably, nucleosome occupancy–derived scores from *RERE* and *SYNPO2* distinguished recurrent from primary cancer with high accuracy (area under the curve = 0.826). Using a machine-learning approach, integration of these cfDNA features accurately predicted breast cancer relapse. Collectively, these findings demonstrate that cfDNA-based profiling focused on transcriptional alterations provides a sensitive strategy for detecting breast cancer recurrence.

**Significance:**

cfDNA-based (epi)genomic profiling captures transcriptionally regulated chromatin and nucleosome remodeling during the acquisition of therapy resistance and relapse, enabling minimally invasive, mechanistically informed detection of breast cancer recurrence. Targeting transcriptionally relevant genomic loci provide clinically actionable biomarkers to monitor therapy resistance and guide precision treatment decisions in real time.

## Introduction

Breast cancer is one of the most common cancers among women worldwide (1). Based on molecular features, including the expression of estrogen receptor, progesterone receptor, and human epidermal growth factor receptor 2 (HER2), breast cancers are classified into distinct clinical subtypes. The majority of patients with early-stage breast cancer can be cured with surgery and radiotherapy in combination with subtype-specific chemotherapy (2). However, a subset of patients develops disease recurrence and therapy resistance, a risk that increases further in advanced stages. Although genomic alterations, such as mutations in *ESR1* or *PIK3CA*, are well-established mechanisms underlying therapy resistance (3–5), nongenetic features, including enhancer reprogramming, also contribute by promoting tumor plasticity through transcriptional transitions (6, 7). Previously, we demonstrated that transcriptional and chromatin dynamics in breast cancer cells emerge during the acquisition of therapy resistance (8, 9). Genomic regions undergoing such transcriptional alterations may therefore represent promising targets for monitoring therapy resistance and disease recurrence.

Cell-free DNA (cfDNA), which contains tumor-derived genome released into the circulation, has emerged as a promising clinical tool for detecting subtle changes indicative of early disease, monitoring disease progression, evaluating therapeutic efficacy, and predicting prognosis. Recently, various cfDNA-based approaches have successfully identified cancer-associated signals. Beyond genomic alterations, transcriptomic and epigenetic states are also reflected in cfDNA fragmentation pattern, enabling improved prediction of disease relapse prior to conventional clinical diagnosis (10). Consequently, cfDNA-based liquid biopsy technologies hold substantial potential for the personalization of cancer treatment. Moreover, expanding analyses of epigenetic cfDNA features, such as DNA methylation, fragmentation pattern, and DNA topology, has significantly broadened the clinical utility of cfDNA biology. In this study, focusing on genomic regions that undergo transcriptional alterations in therapy-resistant breast cancer models (8), we used a targeted sequencing approach to identify cfDNA features associated with recurrent and/or metastatic breast cancer (mBC) and develop novel liquid biopsy markers for detecting breast cancer relapse. In addition, we analyzed noncoding genomic variation, copy-number alterations (CNA), and nucleosome landscapes linked to the metastatic state. Finally, we applied a multimodal model incorporating nucleosome profiling to predict patients harboring recurrent breast cancers.

## Materials and Methods

### Patients and samples

**Figure 1.**
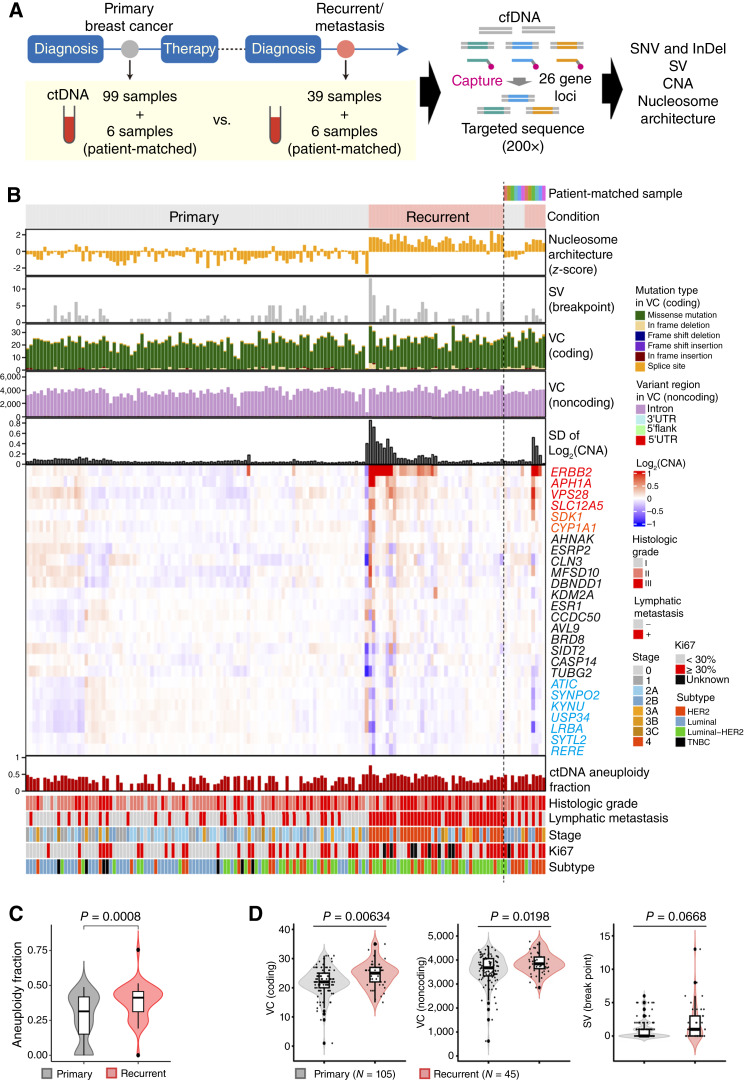
Targeted cfDNA sequencing to assess nucleosome architecture, genomic variants, and CNAs. **A,** Schematic overview of study design and analytic workflow. InDel, insertion and deletion; SNV, single-nucleotide variation. **B,** Per-sample overview of nucleosome features, genomic variants, CNAs, tumor fraction, and clinical information. Each vertical line indicates an individual patient sample, and each horizontal line indicates the analyzed data, as shown by the corresponding color scheme on the right. ctDNA aneuploidy fraction and VC, SD of CNV, and nucleosome architecture are shown in **C** and **D**, Figs. 2 and 3, respectively. TNBC, triple-negative breast cancer. **C,** The ctDNA aneuploidy fraction in cfDNA estimated using *ichorCNA* in primary (*N* = 105; gray) and recurrent (*N* = 45; pink) samples. **D,** Comparison of VC coding and noncoding regions and SVs detected in primary samples (*N* = 105) vs. recurrent samples (*N* = 45).

A retrospective cohort of 150 blood samples was collected from 99 patients with primary breast cancer (pBC; 105 samples) and 34 patients with recurrent and/or mBC (45 samples) who were diagnosed and treated at Kumamoto University Hospital between 2007 and 2017 (Fig. 1A; Table 1; Supplementary Fig. S1; Supplementary Table S1). Among the 105 samples from the 99 patients with pBC, 99 were collected before initial treatment, and six were also collected after the neoadjuvant setting independent of each subtype. The 45 samples from the 34 patients with mBC were collected at time point of recurrence and/or distant metastasis, as confirmed by clinical examinations. Of these patients, six had samples available from both pBC and mBC (Fig. 1A; Supplementary Fig. S1). The procedures of blood collection and DNA isolation have been described previously (5, 11).

**Table 1.** Sample characteristics and clinical information.

Sample information	Primary *n* = 105	Recurrent/metastatic *n* = 45	*P* value
Age (years)	​	​	​
Mean	62.57	60.64	0.396
Range	30–95	33–86	​
Estrogen receptor	​	​	​
Positive	76 (72.4%)	30 (66.7%)	0.4811
Negative	29 (27.6%)	15 (33.3%)	​
Progesterone receptor	​	​	​
Positive	61 (58.2%)	19 (41.2%)	0.1075
Negative	44 (41.9%)	26 (57.8%)	​
HER2	​	​	​
Negative	56 (53.3%)	4 (11.1%)	**<0.0001**
Positive	49 (46.7%)	41 (88.9%)	​
Subtype	​	​	​
Luminal	49 (46.7%)	4 (8.9%)	​
Luminal–HER2	27 (25.7%)	26 (57.8%)	​
HER2	22 (21%)	15 (33.3%)	​
Triple-negative breast cancer	7 (6.7%)	0 (0%)	​
Ki67	​	​	​
<30%	71	13	​
≥30%	33	24	​
Unknown	1	8	​
Histologic grade	​	​	​
I and II	76 (72.4%)	20 (44.4%)	**0.0015**
III	29 (27.6%)	25 (55.6%)	​
Clinical stage at diagnosis	​	​	​
0	3	0	​
I	34	1	​
II	54	10	​
III	14	5	​
IV	0	29	​
Tumor size	​	​	​
T <2 cm	49 (46.7%)	11 (24.4%)	​
T ≥2 cm	56 (53.3%)	10 (22.2%)	​
unknown	0	24 (53.3%)	​
Lymph node metastasis	​	​	​
Negative	75 (71.4%)	7 (15.6%)	**<0.0001**
Positive	30 (28.6%)	38 (84.4%)	​

Statistically significant values in bold.

This study was conducted according to the principles of the Declaration of Helsinki and was approved by the Institutional Ethics Committee of the Kumamoto University (approval number 492).

### Library preparation and sequencing

Sequencing libraries were prepared from 15 to 30 ng of plasma DNA per sample using KAPA Hyper Prep Kit (Kapa Biosystems) and xGen Duplex Seq Adapter-Tech Access [Integrated DNA Technologies (IDT)]. Hybrid capture was performed using a custom-designed panel comprising 29,551 probes covering 4.9 Mb (NGS Discovery Pools prepared by IDT), targeting the following genomic loci: *RERE*, *APH1A*, *AHNAK*, *SYTL2*, *CYP1A1*, *CLN3*, *ESRP2*, *DBNDD1*, *USP34*, *MFSD10*, *LRBA*, *BRD8*, *VPS28*, *KDM2A*, *SIDT2*, *ERBB2*, *TUBG2*, *CASP14*, *KYNU*, *ATIC*, *SLC12A5*, *CCDC50*, *SYNPO2*, *ESR1*, *SDK1*, and *AVL9* (Supplementary Table S2; Supplementary Fig. S1). Libraries were indexed, pooled, and sequenced on the NovaSeq using 100-bp paired-end sequencing reads (2 × 100 bp) to achieve a target depth of 200× coverage.

### Targeted library alignment and mutation calling

Sequence reads were aligned to human genomic reference sequences (GRCh37) using the Burrows–Wheeler aligner (version 0.5.9-r16; Wellcome Trust Sanger Institute) and deduplicated using *rmdup* function in *SAMtools* (v0.1.18). Somatic single-nucleotide variants and insertions/deletions were identified using the *MuTect2* in the Genome Analysis Toolkit (GATK; version 3.8–0) following GATK best practices (Broad Institute of MIT and Harvard) in tumor-only mode. Variant calls with a total read depth <10 were excluded prior to downstream analyses. Detected somatic variants were functionally annotated and converted to Mutation Annotation Format (MAF) files using the *Funcotator* tool in GATK (v4.2.6.1). MAF data were subsequently analyzed and visualized using the *maftools* package (v2.12.0; Supplementary Table S3).

### Estimation of circulating tumor DNA aneuploidy fraction using *ichorCNA*

The *ichorCNA* framework estimates the circulating tumor DNA (ctDNA) tumor fraction by modeling CNAs derived from low-coverage whole-genome sequencing (WGS) data of cfDNA (12). Previous studies have shown that sequencing reads falling outside targeted regions can serve as a surrogate for low-coverage WGS, enabling genome-wide copy-number profiling and subsequent estimation of tumor fraction (13, 14). In the present study, reads mapping to targeted regions were excluded, and only off-target reads were retained for downstream analysis. These reads were processed using the *ichorCNA_offtarget* pipeline (script version dated January 6, 2020) with default settings to infer tumor fraction. We defined the *ichorCNA-*inferred tumor fraction as the ctDNA aneuploidy fraction in this study. For each sample, the estimate corresponding to the highest-likelihood solution was selected. Correlations between variables were assessed using Spearman’s rank correlation coefficients.

### Fragment size analysis

Fragment size distributions were analyzed and visualized using the *ctDNAtools* (v0.4.1) package. The fragment ratio was calculated as the number of short fragments (100–140 bp) divided by the number of long fragments (141–220 bp) within each 100-bp genomic bin, and comparisons between groups were performed using the Wilcoxon matched-pairs signed-rank test.

### Quantification of cfDNA coverage across genomic loci

Coverage counts across the 26 genomic loci were generated using the bedtools multicov function (v.2.17.0) and subsequently normalized by genomic length (read count per kilobase; length normalization), mean counts per sample (sample normalization), and mean counts per genomic locus (location normalization). To evaluate alteration in cfDNA coverage between primary and recurrent/metastatic samples, fragmentation profiles at each genomic locus were compared between the two groups using the Welch two-sample *t* test implemented in RNAseqChef (15). For each sample, the standard deviation (SD) of cfDNA coverage alterations across all targeted gene loci was calculated, and these values were compared between the primary and recurrent/metastatic groups using the Welch *t* test.

### Structural variant calling and breakpoint quantification using *GRIDSS2*

Structural variants (SV) were called from cfDNA-targeted sequencing data using *GRIDSS2* (v2.13.2). Sequence reads were aligned to the human reference genome (GRCh37) as described above, and PCR duplicates were removed prior to SV analysis. As a normal reference, three cfDNA sequencing datasets from young healthy controls (GSE114511, replicates 1–3) were merged to generate a pooled normal BAM file, which was provided to GRIDSS2 together with the tumor cfDNA BAM files. The standard *GRIDSS2* pipeline was executed using default parameters. Variant calls were generated in VCF format and imported into R for downstream processing using the *StructuralVariantAnnotation* package (v1.12.0). The imported data were filtered to remove variant calls with a QUAL score <1,000 prior to further analysis. In this cfDNA dataset, all retained calls were annotated as SVTYPE = “smallNoSR,” representing small structural events without split-read support under the *GRIDSS2* classification scheme. Filtered calls were summarized for each sample as a quantitative measure of SV burden.

### cfDNA nucleosome footprinting

To assess nucleosome occupancy in cfDNA, a per-base windowed protection score (WPS) was calculated as previously described (16). Briefly, for each genomic position, the WPS was defined as the number of fragments completely spanning a 120-bp window minus the number of fragment endpoints within the same window and was computed using the R package *ctDNAtools* (v0.4.1) with default setting (17). For each locus, WPS-based nucleosome footprints were statistically compared between primary and recurrent/metastatic samples using *limma* (v3.52.4), and *P* values were adjusted for multiple testing using the Benjamini–Hochberg procedure. For each sample, a composite nucleosome score was defined as the sum of WPS values across loci with increased nucleosome occupancy minus the sum across loci with decreased occupancy [an adjusted *P* value (*P*adj) < 0.3]. Receiver operating characteristic (ROC) curves were constructed, and the corresponding area under the curve (AUC) values were calculated using the *pROC* package (v1.18.4) using default settings.

### Construction and validation of a recurrence prediction model

To construct cfDNA-based signatures of tumor recurrence, LASSO-penalized logistic regression models were fitted with recurrence status (primary vs. recurrence) as the binary outcome. Predictors consisted of normalized WPS values at candidate loci selected by differential WPS analysis (FDR <0.5), optionally combined with additional cfDNA-derived features such as estimated ctDNA aneuploidy fraction, CNAs, variant counts (VC), and SVs. All continuous predictors were centered and scaled to unit variance prior to model fitting. Penalized logistic regression was implemented using the *glmnet* algorithm with an L1 penalty (α = 1). For the patient-matched validation analysis, samples from six patients with paired primary and recurrent/metastatic cfDNA (*n* = 12) were held out *a priori* as an independent test set, and the remaining 138 samples were used as the training cohort. The optimal regularization parameter (λ) was selected by fivefold stratified cross-validation within the training data, after which the final model was refitted on all 138 training samples and applied once to the 12 held-out patient-matched samples. Model discrimination was evaluated using ROC curves, and the corresponding AUC values were calculated using the DeLong method. A one-sided Wilcoxon signed-rank test was used to assess whether predicted recurrence probabilities were higher in recurrent than in primary samples.

## Results

### Overview of targeted deep sequencing of cfDNAs in clinical breast cancers

To achieve early detection of tumor recurrence and metastasis, we selected 26 gene loci whose transcriptional levels were dynamically altered in therapy-resistant models of human breast cancer cells in our previous report (Fig. 1A; Supplementary Fig. S1; ref. 8). We designed capture probes to enable enriched sequencing not only of exonic regions but also of intronic regions, 5′ upstream/flanking regions, and 3′ downstream regions, thereby facilitating the detection of diverse genomic alterations. The total targeted genomic region spanned approximately 4.9 megabases (Mb), and probe coverage across loci ranged from 56% to 93% (Supplementary Table S2). We then performed targeted deep sequencing of cfDNA (median depth, ∼200×) from 105 blood samples obtained from 99 patients with pBC and from 45 blood samples obtained from 34 patients with recurrent and/or mBC (Fig. 1A; Table 1; Supplementary Fig. S1; Supplementary Table S1). All patients provided at least one cfDNA sample, and 20 patients contributed multiple samples. As shown in Supplementary Fig. S1A, longitudinal sampling included six patients with pBC sampled before and after chemotherapy, nine patients with mBC serially during progression across different lines of therapy, and six patients with matched samples from primary and recurrent states (Fig. 1A; Supplementary Fig. S1).

In this study, the proportion of tumor-derived DNA in blood samples, defined as the ctDNA aneuploidy fraction, had a median value of 0.26 (range, 0–0.49) in pBC and 0.39 (range, 0–0.75) in mBC (Fig. 1B). As previously described, the ctDNA aneuploidy fraction was significantly higher in recurrent samples than in primary samples (*P* < 0.0008, Wilcoxon test; Fig. 1C). Notably, the proportion of samples with an undetectable ctDNA aneuploidy fraction was 25% (26/105) in pBC, whereas only 2% (1/45) of mBC samples showed undetectable levels, consistent with previous reports (12, 14) and supporting the validity of this study. The median cfDNA mutation counts were 23 per sample (range, 1–35) in the coding regions and 3,733 per sample (range, 620–4,771) in the noncoding regions, including introns. These values remained significantly higher in recurrent samples than in primary samples (*P* = 0.00634 and *P* = 0.0198, respectively; Mann–Whitney *U* test; Fig. 1B and D), suggesting that the observed differences are not solely attributable to variations in ctDNA abundance. Among the 26 gene loci examined, 10 loci—*AVL9*, *CCDC50*, *DBNDD1*, *KDM2A*, *RERE*, *SDK1*, *SLC12A5*, *SYNPO2*, *TUBG2*, and *USP28*—exhibited significantly higher VCs in recurrent samples than in primary samples (*CCDC50* and *SYNPO2*, *P* < 0.006, remaining eight loci, *P* < 0.05; Wilcoxon test; Fig. 1B; Supplementary Fig. S2), indicating locus-specific enrichment beyond global ctDNA levels. SV breakpoints were detected at a median of 0.5 per sample (range, 0–13) and showed a trend toward increased frequency in recurrent samples (*P *= 0.0668, Mann–Whitney *U* test; Fig. 1D), further supporting qualitative differences in genomic alteration patterns.

The mutation profiles of coding and noncoding regions are summarized in Supplementary Fig. S3A–S3C and Supplementary Table S3. In coding regions, most variants were missense mutations; however, synonymous variants were excluded from this analysis. The majority of variant types were single-nucleotide polymorphisms (SNP; 92.9%), with smaller proportions of insertions (0.85%) and deletions (6.27%). Among substitutions types, T > C (49.7%) and C > T (25.9%) were the most frequent (Supplementary Fig. S3B; Supplementary Table S3). The top 10 genes by VC were detected in more than 75% of samples in this study. Comparison of variant frequencies between pBC and mBC revealed significant enrichment of variants in the *MFSD10*, *KDM2A*, and *ESRP2* loci in mBC, with odds ratios of 8.03, 12.76, and 2.76, respectively (Supplementary Fig. S4A). Lollipop plots for these three genes illustrated the positions of variants and corresponding amino acid substitutions enriched in mBC (Supplementary Fig. S4B). Notably, five of the seven variants enriched in mBC were very rare coding variants (gnomAD allele frequency <0.01), suggesting a potential association with an increased risk of recurrence or metastasis (Supplementary Fig. S4C).

Also in noncoding regions, the most frequent variant types were SNPs (88.7%), followed by deletions (6.72%) and insertions (4.60%), with the majority of substitutions being T > C (32.8%) and C > T (33.8%; Supplementary Fig. S3C; Supplementary Table S3). Thirteen probes harbored significantly more frequent variants in mBC than in pBC (Supplementary Fig. S5A and S5B). Our previous study demonstrated that 12 of 13 genomic loci, including *SDK1*, *LRBA*, *ESR1*, *RERE*, *CCDC50*, *KDM2A*, *SYNPO2*, *USP34*, *AHNAK*, *AVL9*, *KYNU*, and *SYTL2*, were dynamically regulated at both the mRNA level and in adjacent noncoding RNAs in therapy-resistant breast cancer models (8). Collectively, these results indicate that our targeted sequencing approach is sufficient to detect variants that discriminate between mBC and pBC.

### Shorter cfDNA fragments are enriched in mBC

**Figure 2.**
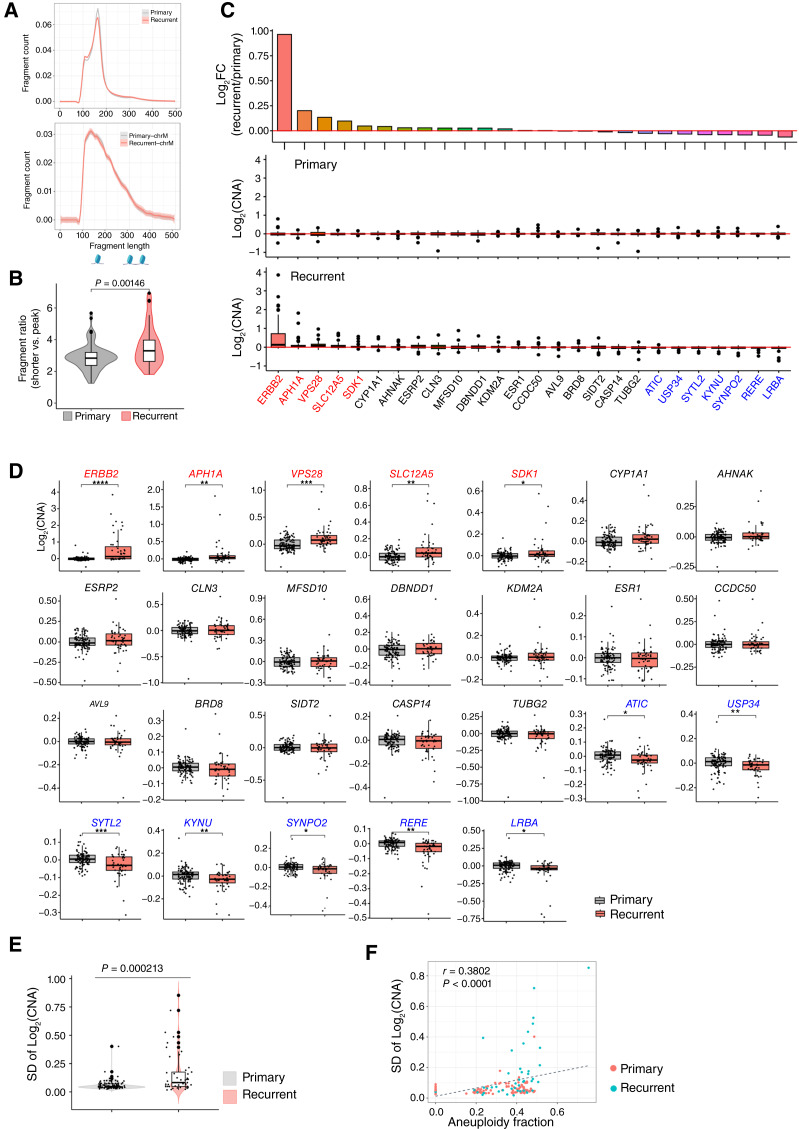
Distribution of fragment lengths and cfDNA coverage across 26 genomic loci. **A,** Fragment length frequency distribution of genomic (top) and mitochondrial (bottom) fraction from primary (gray) and recurrent (pink) samples. **B,** Comparison of fragment ratios of targeted genes in relation to CNA, calculated as the ratio of fragments shorter than 140 bp to peak fragment counts, in primary (*N* = 105; gray) and recurrent (*N* = 45; pink) samples. **C,** Quantification of cfDNA coverage across the 26 genomic loci. **D,** Comparison of cfDNA coverage of targeted genes between primary and recurrent/metastatic samples. Asterisks denote statistical significance with Welch’s *t* test. *, P < 0.05; **, *P* < 0.005; ***, *P* < 0.0005; ****, *P* < 0.0001 for the indicated comparison. **E,** Comparison of the SD of CNAs between primary (*N* = 105; gray) and recurrent/metastatic (*N* = 45; pink) samples. **F,** Relationship between CNAs and ctDNA aneuploidy fraction assessed by Spearman’s correlation in primary (*N* = 105; pink) and recurrent (*N* = 45; blue) samples.

Because cfDNA fragment length has been reported to be associated with tumor-derived variants (18, 19), we examined the length distribution of genomic and mitochondrial cfDNA fractions in Fig. 2A and B (top and bottom, respectively). In the genomic fraction, the proportion of shorter cfDNA fragments (<140 bp) was significantly higher in recurrent samples than in primary samples. This finding is consistent with our observation that genomic VCs were significantly higher in recurrent cases than in primary samples (Fig. 1B and D).

### Recurrent breast cancer is characterized by cfDNA copy-number aberration profiles

As cfDNA molecules are primarily nucleosome-associated, their fragmentation patterns have been reported to reflect gene regulatory states (16, 20). Condensed chromatin is generally protected from the action of various endonucleases, whereas open chromatin regions are more susceptible to such degradation. To assess CNAs, we quantified cfDNA coverage across the 26 targeted genomic loci. As shown in Figs. 1B and 2C–E, recurrent samples exhibited significantly greater variability in read counts than primary samples. Increased copy number at gene loci such as *ERBB2*, *APH1A*, and *VPS28*, which are frequently amplified in breast cancers (21, 22), was consistently observed in recurrent samples, in agreement with amplification patterns reported in clinical breast cancers in The Cancer Genome Atlas database (Supplementary Fig. S6). In addition to these gains, significantly decreased copy number at loci such as *LRBA*, *RERE*, and *SYNPO2* was also observed in recurrent samples. Regions showing reduced fragmentation in recurrent samples are thought to reflect more accessible chromatin states than those in primary samples (16, 20). Because the recurrent cohort included a higher proportion of HER2-positive cases, we performed a subtype-stratified analysis focusing on HER2-positive samples (*N* = 90; primary *N* = 49 and recurrent *N* = 41). As shown in Supplementary Fig. S7A and S7B, cfDNA copy-number profiles remained more variable in recurrent disease than in pBC, consistent with the findings from the overall cohort. Furthermore, the ctDNA aneuploidy fraction was significantly correlated with CNA burden (Spearman’s ρ = 0.3802, *P* < 0.0001; Fig. 2F), suggesting that cfDNA fragmentation and coverage alterations reflect variation in tumor-derived DNA levels in mBC samples. Collectively, these results indicate that cfDNA fragmentation and copy-number profiles exhibit increased variability in recurrent disease compared with pBC, reflecting both quantitative and qualitative differences in tumor-derived signals, independent of HER2-positive subtype enrichment.

### Detection of breast cancer recurrence using nucleosome architecture

**Figure 3.**
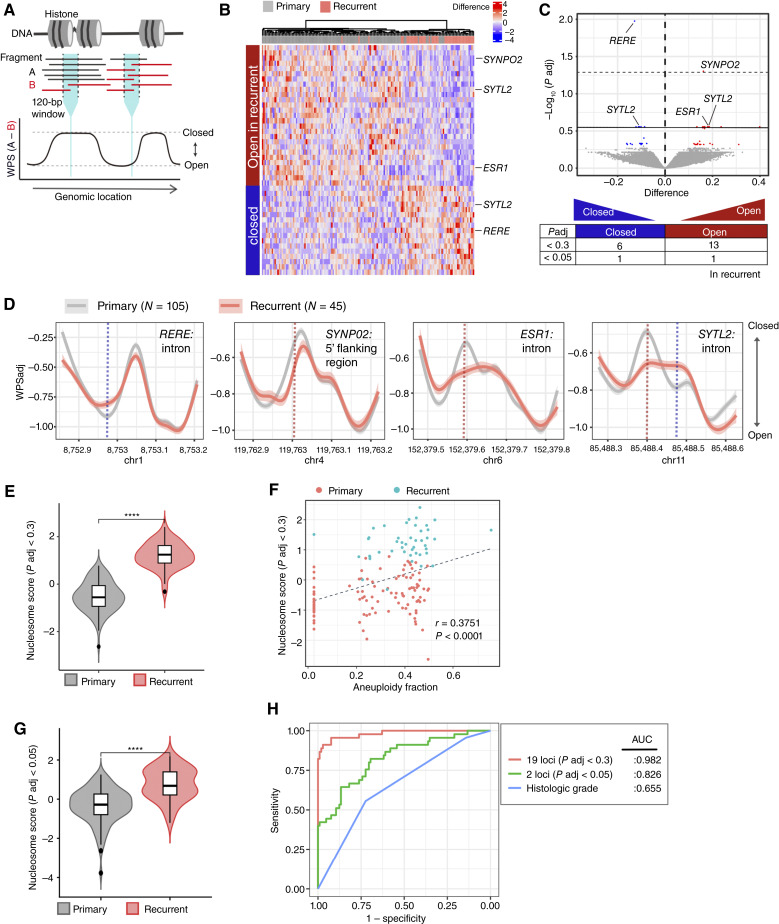
Detection of recurrence and metastasis in patient samples using targeted cfDNA nucleosome profiling. **A,** Schematic illustration of nucleosome occupancy based on a per-base WPS, calculated by subtracting the number of fragment endpoints within a 120-bp window (indicated by B) from the number of fragments completely spanning the window (indicated by A). **B,** Hierarchical clustering of cfDNA WPS value at 44 genomic sites that were differentially altered (Wilcoxon rank-sum test, *P*adj < 0.5), based on the log_2_ FC between primary (gray) and recurrent (pink) samples. **C,** Volcano plots showing differential nucleosome occupancy based on cfDNA WPS values, plotted as log_2_FC vs. −log_10_ (*P*adj). Solid and dashed horizontal lines indicate *P*adj thresholds of 0.3 and 0.05, respectively. Representative genomic loci, including the top four sites with open or closed nucleosomes in the ranked list (Supplementary Table S4), are indicated. **D,** Mean adjusted WPS values for primary (*N* = 105; gray) and recurrent (*N* = 45; pink) samples at the top four sites indicated in **C**. **E,** Comparison of nucleosome score calculated as *z*-scores of WPS across 19 genomic sites (*P*adj < 0.3) between primary (*N* = 105) and recurrent (*N* = 45) samples. Asterisks denote statistical significance with Mann-Whitney *U* test (****, *P* < 0.0001). **F,** Relationship between nucleosome scores and ctDNA aneuploidy fraction assessed by Spearman’s correlation in primary (*N* = 105; pink) and recurrent samples (*N* = 45; blue). **G,** Comparison of nucleosome scores calculated as *z*-scores of WPS at the top two genomic loci (*P*adj < 0.05), including the *SYNPO2* locus and two 10-nt windows upstream and downstream, between primary (*N* = 105) and recurrent (*N* = 45) samples. Asterisks denote statistical significance with Mann-Whitney *U* test (****, *P* < 0.0001). **H,** ROC curves of nucleosome scores and histologic grade in primary (*N* = 105) and recurrent (*N* = 45) samples. The 95% confidence intervals for the AUC are shown.

Given the presence of variable genomic loci observed in recurrent samples compared with primary samples, we assessed nucleosome occupancy using *ctDNAtools* (https://alkodsi.github.io/ctDNAtools/index.html; ref. 17). Nucleosome occupancy was quantified using a per-base WPS (16) calculated by subtracting the number of fragment endpoints within a 120-bp window from the number of fragments completely spanning the same window (Fig. 3A). High WPS values indicate increased protection of DNA from enzymatic digestion, whereas low values indicate reduced protection. To characterize WPS features, we identified 44 genomic sites exhibiting differential variation between recurrent and primary samples [Wilcoxon rank-sum test, adjusted *P* value (*P*adj) < 0.5] using a permissive threshold to capture a broad set of candidate loci for downstream refinement. These sites were subsequently classified into open and closed chromatin states based on their log_2_ fold change (FC) between two sample groups (Fig. 3B; Supplementary Tables S4 and S5). As shown in Fig. 3C, volcano plots of WPS values plotted as log_2_FC versus −log_10_ (*P*adj) illustrate genomic sites with differential nucleosome occupancy. Representative WPS profile for the top four sites exhibiting open or closed nucleosome states in the ranked list are shown in Fig. 3D and Supplementary Table S4. To assess nucleosome dynamics, we calculated *z*-scores for WPS across 19 genomic sites (*P*adj < 0.3), applying an intermediate threshold to retain loci with moderate statistical support for downstream analysis. Nucleosome scores were standardized using their mean and SD. Recurrent samples exhibited significantly increased variability in WPS compared with primary samples (Figs. 1B and 3E). In addition, nucleosome scores were significantly correlated with ctDNA aneuploidy fractions (Spearman’s ρ = 0.3751, *P* < 0.0001), indicating that these 19 genomic sites reflect, at least in part, variation in tumor-derived DNA levels in cfDNA samples (Fig. 3F). To further refine genomic sites showing differential nucleosome occupancy between recurrent and primary samples, the top two loci, *RERE* and *SYNPO2*, were identified using a more stringent threshold (*P*adj < 0.05). Nucleosome scores derived from two genomic loci spanning 260 nucleotides—including the *SYNPO2* site and two adjacent genomic windows of 10 nucleotides upstream and downstream (Supplementary Tables S4 and S5)—demonstrated distinct nucleosome occupancy patterns between primary and metastatic samples (Fig. 3G). Given the subtype imbalance noted above, we further evaluated these patterns within the HER2-positive subset. Significant differences in nucleosome occupancy were consistently observed (Supplementary Fig. S7C and S7D), indicating that these signals are not solely driven by differences in subtype composition. To evaluate whether nucleosome *z*-scores could discriminate mBC from pBC samples, ROC curves were generated, and AUC values were calculated to assess sensitivity and specificity. As shown in Fig. 3H, the use of either 19 loci or only 2 loci yielded AUC values ranging from 0.826 to 0.982 for distinguishing mBC from pBC, markedly outperforming the AUC of histologic grade (0.655), a currently established prognostic factor.

### Multimodal cfDNA analysis improves detection of breast cancer recurrence

**Figure 4.**
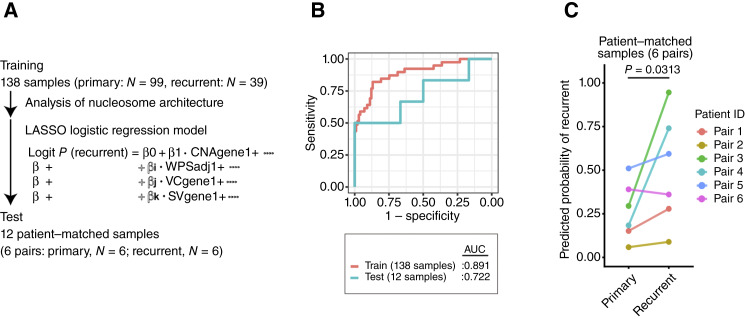
Multimodal integration model for predicting breast cancer recurrence. **A,** Strategy for integrating multimodal cfDNA features using a LASSO logistic regression model. **B,** Comparison of ROC curves from multimodal analysis in the training set (*N* = 138; pBC = 99, mBC = 39) and the testing set consisting of patient-matched samples (*N* = 12; pBC = 6, mBC = 6). The 95% confidential intervals for the AUC are shown. **C,** Comparison of recurrence score in six patient-matched primary (pBC) and metastatic (mBC) samples.

Although we confirmed the discriminative ability of the nucleosome scores at these genomic sites in six patient-matched pBC and mBC samples, exclusion of these samples reduced statistical sensitivity while preserving the overall trend. To improved sensitivity, we applied a machine-learning approach and established a cfDNA-based recurrence prediction model by integrating nucleosome scores and other genetic features, including VCs and CNAs (Fig. 4A). In the training cohort, 138 samples (pBC = 99, mBC = 39) were used to construct a classifier based on the least absolute shrinkage and selection operator (LASSO), whereas the remaining 12 patient-matched samples (pBC = 6 and mBC = 6) served as an independent test set to evaluate model performance. As shown in Fig. 4B, the recurrence detection model generated ROC curves, yielding an AUC of 0.891 in the training set and 0.722 in the test subset. This cfDNA-based recurrence prediction model correctly identified recurrence in five of the six patients (Fig. 3C). The only case (pair 6) in which recurrence was not detected involved a patient with brain metastasis, in whom ctDNA is often undetectable in plasma due to the blood–brain barrier (23).

Collectively, these results demonstrate that nucleosome features informed by transcriptional dynamics, particularly when integrated with complementary genomic signals, enable reliable detection of recurrent/mBC.

## Discussion

In this study, we performed targeted sequencing of 150 cfDNA samples derived from liquid biopsy, including 105 primary and 45 recurrent and/or mBCs, focusing on 26 genomic loci transcriptionally regulated during therapy resistance. We demonstrated that somatic variants increased significantly in metastatic cancers not only within coding but also in noncoding regions (Fig. 1). Notably, 13 probes more frequently harbored variants in metastasis than in primary samples, and 12 of these 13 loci were associated with the upregulation of noncoding RNAs derived from neighboring protein-coding genes (Supplementary Fig. S5A and S5B). Because these loci are reportedly longer than genes lacking coordinated regulation of ncRNAs and mRNAs (8), DNA damages induced by anticancer therapy may stochastically escape DNA repair (24), leading to the accumulation of genome damage associated with metastasis. Given that chromatin compaction generally acts as a physical barrier against genotoxic stresses (25), it is plausible that open chromatin states maintained by coordinated ncRNA–mRNA regulation increase genetic vulnerability, thereby promoting the accumulation of somatic variants during anticancer therapy.

Our data also highlight the importance of the nucleosome profiling, rather than genetic alterations alone, for detecting breast cancer relapse. cfDNA fragmentation profiles targeting transcriptionally dynamic loci during anticancer therapy were highly variable in recurrent disease but remained relatively consistent in primary samples (Fig. 2). Furthermore, our assessment of nucleosome occupancy revealed that nucleosome scores derived from either 2 or 19 specific loci, spanning genomic regions of 260 or 1,820 nt, respectively, could distinguish relapse from primary samples (Fig. 3). Recently, cfDNA fragmentation patterns have emerged as a means to infer epigenomic and transcriptomic information (10, 20, 26–28). However, most existing studies rely on WGS, which is not cost-effective for the identification of clinically actionable biomarkers. To our knowledge, this study represents the first targeted nucleosome analysis—including noncoding regions—that has been validated for the diagnosis of metastatic cancers using cfDNA. The identified regions therefore hold strong potential as simple, cost-effective markers for clinical translation. Although genome-wide nucleosome profiling of cfDNA has been reported to predict cancer subtypes (26), we did not observe clear subtype-specific differences in our dataset. In addition, this targeted sequencing approach did not reliably detect activating *ESR1* mutations associated with endocrine resistance, such as Y537S and D538G (3–5), in recurrent samples (Supplementary Table S3). An important limitation of this study is the imbalance in molecular subtypes, particularly the higher proportion of HER2-positive cases compared with that typically observed in the general breast cancer population. Given that HER2 amplification is associated with increased genomic instability, this enrichment may have influenced the observed cfDNA copy number and nucleosome patterns. Although subtype-stratified analyses within the HER2-positive subset recapitulated the main findings (Supplementary Fig. S7), these analyses do not fully exclude potential confounding effects arising from subtype-specific biology. Furthermore, the retrospective design and the use of largely unmatched primary and recurrent samples may introduce additional heterogeneity that could affect the interpretation of the results. Therefore, validation in larger, prospective cohorts will be required to improve subtype-specific relapse prediction and confirm the generalizability and clinical utility of this approach.

## Supplementary Material

Supplementary Table S1 Patients’ characteristics and clinical information.

Supplementary Table S2 Probe information used for deep sequencing

Supplementary Table S3 Somatic variants analyzed using the maftools

Supplementary Table S4 WPS classification by log_2_ fold change between recurrent and primary samples

Supplementary Table S5 44Forty-four genomic sites that exhibited differential variation between recurrent and primary samples

Supplementary Figure S1 Figure S1. Targeted gene information and clinical breast cancer samples used for deep sequencing.

Supplementary Figure S2 Figure S2. Comparison of variant counts in coding and non-coding regions.

Supplementary Figure S3 Figure S3. Overview of variant profiles.

Supplementary Figure S4 Figure S4. Comparison of mutational signatures between primary and recurrent samples.

Supplementary Figure S5 Figure S5. Comparison of non-coding variant sites and their counts between primary and recurrent samples.

Supplementary Figure S6 Figure S6. Oncoprint analysis of the 26 targeted loci in clinical breast cancer samples from the TCGA study.

Supplementary Figure S7 Figure S7. Quantification of cfDNA coverage across 26 genomic loci and nucleosome scores in the HER2-positive subset.

## Data Availability

The targeted cfDNA sequencing datasets have been deposited in the DNA Data Bank of Japan (https://www.ddbj.nig.ac.jp/index-e.html) under the accession number JGAS000812. Other data generated in this study are available upon request to the corresponding author.
