# Supplementary material for: Transcriptionally Informed Nucleosome Profiling of Circulating Cell-Free DNA Predicts Breast Cancer Recurrence
Source: Cancer Res Commun. 2026 Jun 15;6(6):1405–14. doi: 10.1158/2767-9764.CRC-26-0263 (PMC13266714; doi:10.1158/2767-9764.CRC-26-0263)
Supplement: Supplementary Table S1 — Patients’ characteristics and clinical information. [file crc-26-0263_supplementary_table_s1_suppst1.pdf]

Patient information	primary n = 99	recurrent / metastatic n = 34	*P* value
**ER**			
positive	74 (74.7%)	24 (70.6%)	0.635
negative	25 (25.3%)	10 (29.4%)	
**PgR**			
positive	59 (59.6%)	13 (38.2%)	**0.03**
negative	40 (40.4%)	21 (61.8%)	
**HER2**			
negative	56 (53.3%)	4 (11.8%)	**< 0.0001**
positive	43 (46.7%)	30 (88.2%)	
**subtype**			
Luminal	49 (49.5%)	4 (11.8%)	
Luminal-HER2	25 (25.3%)	17 (50.0%)	
HER2	18 (18.2%)	13 (38.2%)	
TNBC	7 (7.1%)	0 (0%)	
**Ki-67**			
< 30%	68	10	
>= 30%	30	18	
unknown	1	6	
**Histological grade**			
I & II	74 (74.7%)	15 (44.1%)	**0.0011**
III	25 (25.3%)	19 (55.9%)	
**Clinical stage at diagnosis**			
0	3	0	
I	33	1	
II	51	7	
III	12	5	
IV	0	21	
**Tumor size**			
T < 2cm	46 (46.5%)	9 (26.5%)	
T >= 2cm	53 (53.5%)	8 (23.5%)	
unknown	0	17 (50.0%)	
**LN metastasis**			
negative	72 (72.7%)	7 (20.1%)	**< 0.0001**
positive	27 (27.2%)	27 (79.4%)	

Supplementary Table S1 Watanabe et al.

Patients' characteristics and clinical information.
