# Supplementary material for: Transcriptionally Informed Nucleosome Profiling of Circulating Cell-Free DNA Predicts Breast Cancer Recurrence
Source: Cancer Res Commun. 2026 Jun 15;6(6):1405–14. doi: 10.1158/2767-9764.CRC-26-0263 (PMC13266714; doi:10.1158/2767-9764.CRC-26-0263)
Supplement: Supplementary Table S5 — 44Forty-four genomic sites that exhibited differential variation between recurrent and primary samples [file crc-26-0263_supplementary_table_s5_suppst5.pdf]

open	probe
chr7:4013926−4014046	*SDK1*
chr4:119762978−119763098	*SYNPO2*
chr4:119762988−119763108	*SYNPO2* *
chr4:119762998−119763118	*SYNPO2* *
chr7:3898076−3898196	*SDK1* *
chr2:61629020−61629140	*USP34* *
chr17:37855069−37855189	*ERBB2*
chr1:8786386−8786506	*RERE*
chr11:85488386−85488506	*SYTL2* *
chr11:85488396−85488516	*SYTL2* *
chr16:28485216−28485336	*CLN3* *
chr17:37852949−37853069	*ERBB2*
chr1:8777386−8777506	*RERE*
chr1:8777396−8777516	*RERE*
chr4:151895449−151895569	*LRBA*
chr1:8596846−8596966	*RERE*
chr7:4235446−4235566	*SDK1* *
chr6:152069219−152069339	*ESR1*
chr3:191082035−191082155	*CCDC50* *
chr4:151643889−151644009	*LRBA* *
chr17:37854279−37854399	*ERBB2*
chr4:119725068−119725188	*SYNPO2*
chr6:152031219−152031339	*ESR1* *
chr6:152379589−152379709	*ESR1* *
chr6:152379599−152379719	*ESR1* *
chr1:8516956−8517076	*RERE*
chr4:151704519−151704639	*LRBA*

closed	probe
chr7:3579006−3579126	*SDK1* *
chr20:44664087−44664207	*SLC12A5*
chr2:61462770−61462890	*USP34*
chr11:85488466−85488586	*SYTL2* *
chr4:119865638−119865758	*SYNPO2*
chr7:4297836−4297956	*SDK1*
chr2:61565480−61565600	*USP34*
chr4:119856318−119856438	*SYNPO2* *
chr1:8752976−8753096	*RERE* *
chr4:151531979−151532099	*LRBA*
chr7:3995906−3996026	*SDK1* *
chr4:151511949−151512069	*LRBA*
chr2:216150214−216150334	*ATIC*
chr2:61630530−61630650	*USP34* *
chr2:143702125−143702245	*KYNU*
chr4:119767618−119767738	*SYNPO2*
chr11:62261928−62262048	*AHNAK*

Supplementary Table S5.
Forty-four genomic sites that exhibited differential variation between recurrent and primary samples.
