# Supplementary material for: Transcriptionally Informed Nucleosome Profiling of Circulating Cell-Free DNA Predicts Breast Cancer Recurrence
Source: Cancer Res Commun. 2026 Jun 15;6(6):1405–14. doi: 10.1158/2767-9764.CRC-26-0263 (PMC13266714; doi:10.1158/2767-9764.CRC-26-0263)
Supplement: Supplementary Figure S1 — Figure S1. Targeted gene information and clinical breast cancer samples used for deep sequencing. [file crc-26-0263_supplementary_figure_s1_suppsf1.pdf]

Designed probes: targeted genes;

▶ *SDK1 RERE LRBA ESR1 USP34 SYNPO2 KDM2A KYNU AVL9 CCDC50 SYTL2 AHNAK ATIC*

▶ *ERBB2 BRD8 SLC12A5 CLN3 SIDT2 DBNDD1 CASP14 ESRP2 TUBG2 CYP1A1 VPS28 APH1A MFSD10*

A

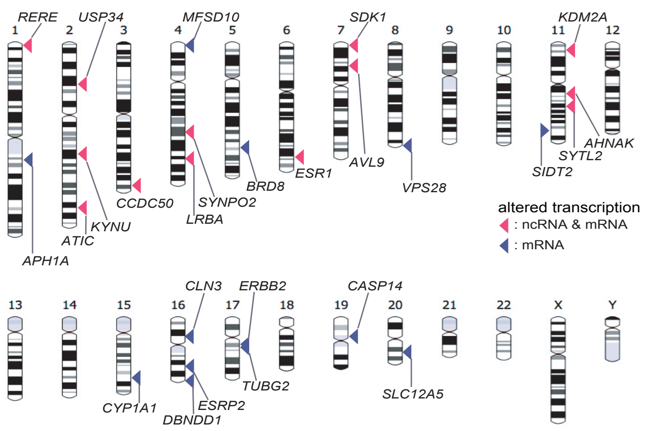

B

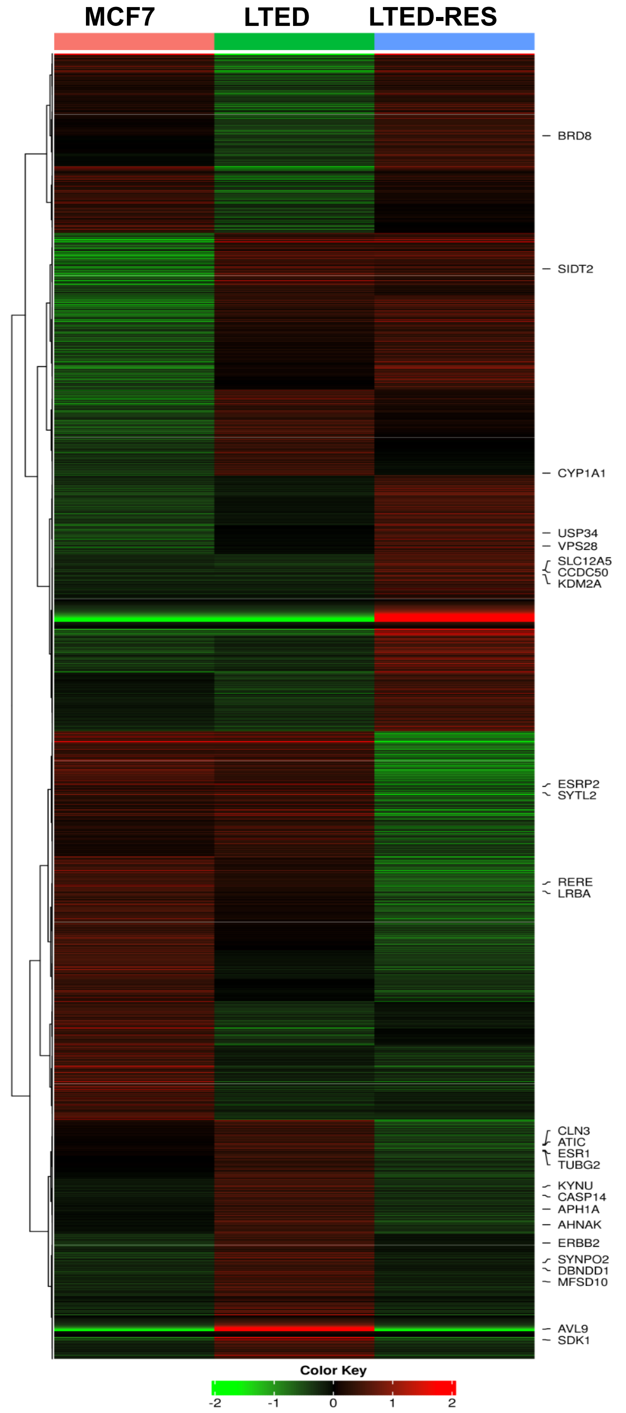

C

150 samples / 127 patients:

- ●primary:105 samples / 99 patients
(12 samles from 6 patients before and after neoadjuvant chemotherapy)
- ●recurrent/metastatic : 45 samples / 34 patients
(20 samples from 9 patients before and after chemotherapy)

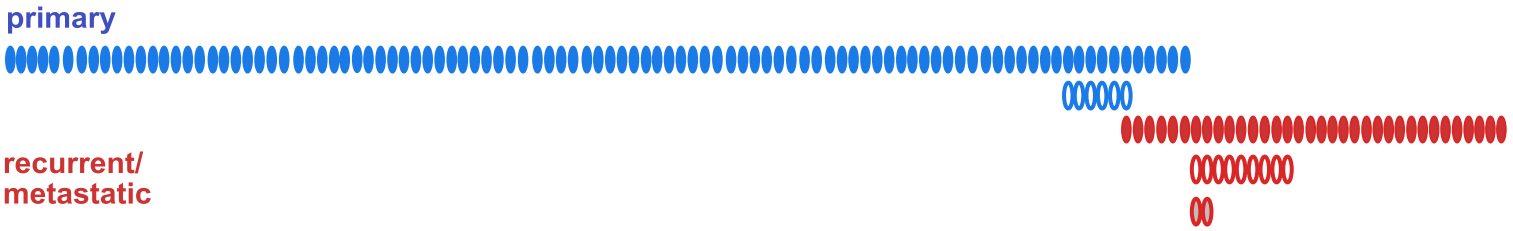

Supplementary Figure S1 Watanabe et al.

Supplementary Figure S1. Targeted gene information and clinical breast cancer samples used for deep sequencing.

(A) Schematic representation of human chromosomes showing the 26 genomic loci selected for probe design. In our previous study (8), we investigated genome-wide transcriptomic changes in endocrine therapy-resistance breast cancer cell models, including MCF7 cells, long-term estrogen-deprived (LTED), and LTED cells treated with resveratrol (LTED-RES). Among the differentially expressed genes (DEGs), 13 genes (pink), such as *ESR1*, showed altered expression coordinated with neighbouring non-coding RNAs (ncRNAs), whereas the remaining 13 genes (blue), such as *ERBB2,* exhibited transcriptional changes independent of ncRNAs. (**B)** Expression levels of the DEGs identified in our previous study (8) were reanalyzed and subjected to hierarchical clustering, using the iDEP.96 tool (http://bioinformatics.sdstate.edu/idep96). The 26 targeted gene loci analyzed in this study are indicated. (**C**) Per-sample overview of patients according to clinical therapy status.
