# Supplementary material for: Transcriptionally Informed Nucleosome Profiling of Circulating Cell-Free DNA Predicts Breast Cancer Recurrence
Source: Cancer Res Commun. 2026 Jun 15;6(6):1405–14. doi: 10.1158/2767-9764.CRC-26-0263 (PMC13266714; doi:10.1158/2767-9764.CRC-26-0263)
Supplement: Supplementary Figure S2 — Figure S2. Comparison of variant counts in coding and non-coding regions. [file crc-26-0263_supplementary_figure_s2_suppsf2.pdf]

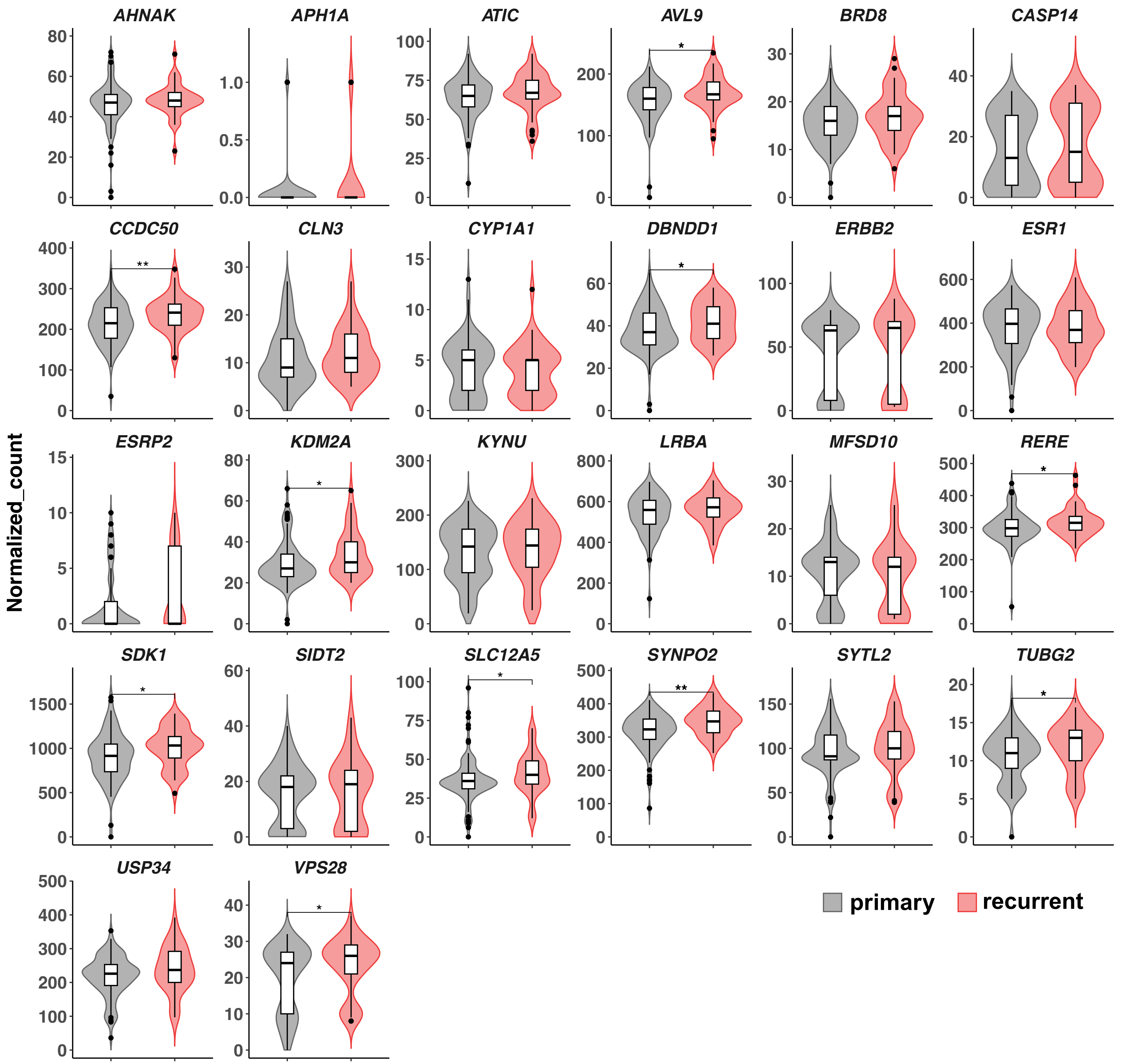

Supplementary Figure S2 Watanabe et al.

Supplementary Figure S2. Comparison of variant counts in coding and non-coding regions.

Comparison of variant counts (VCs) per probe in coding and non-coding regions between primary (N = 105; gray) and recurrent (N = 45; pink) samples.
