# Supplementary material for: Transcriptionally Informed Nucleosome Profiling of Circulating Cell-Free DNA Predicts Breast Cancer Recurrence
Source: Cancer Res Commun. 2026 Jun 15;6(6):1405–14. doi: 10.1158/2767-9764.CRC-26-0263 (PMC13266714; doi:10.1158/2767-9764.CRC-26-0263)
Supplement: Supplementary Figure S3 — Figure S3. Overview of variant profiles. [file crc-26-0263_supplementary_figure_s3_suppsf3.pdf]

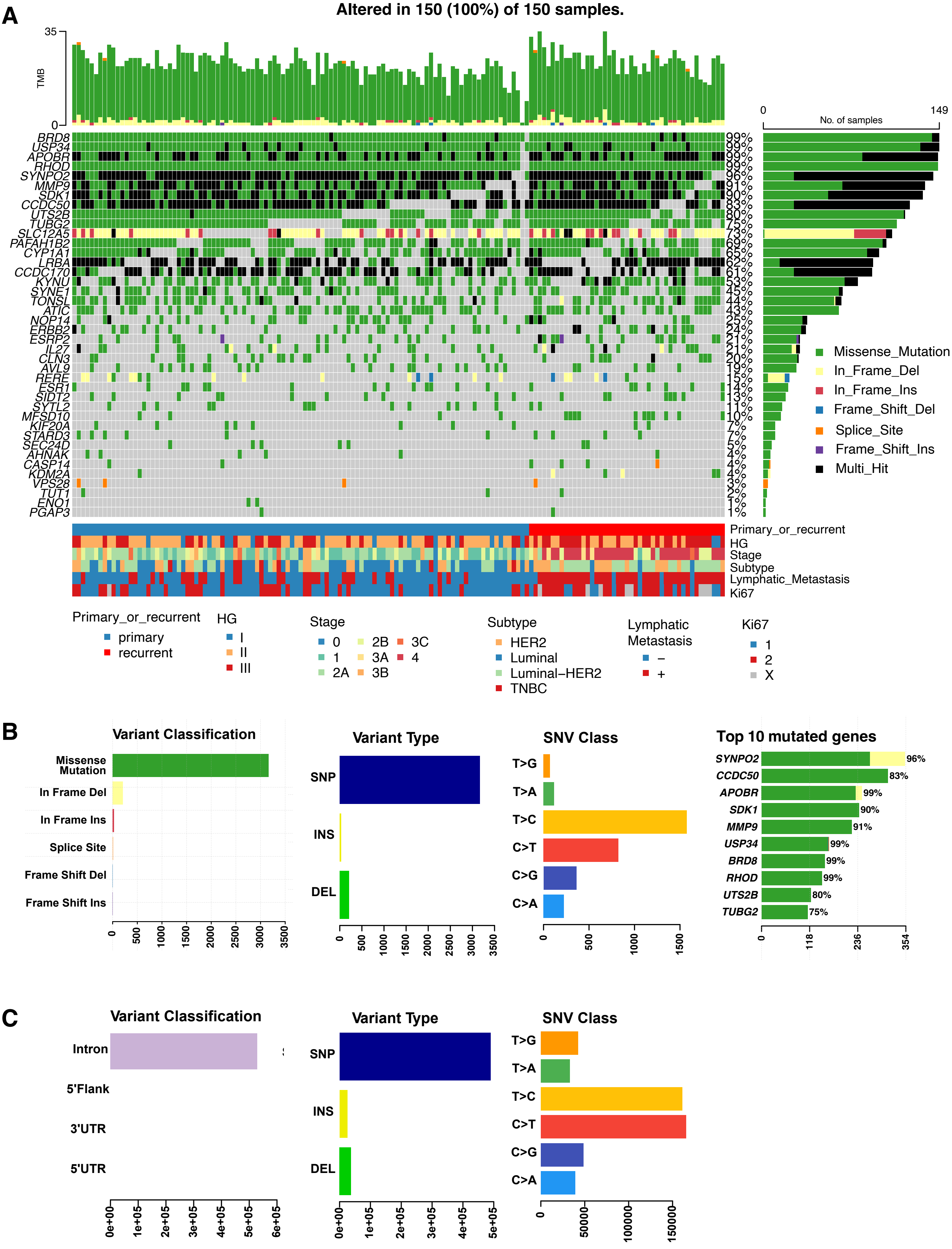

Supplementary Figure S3 Watanabe et al.

Supplementary Figure S3. Overview of variant profiles.

(A) Oncoplot showing all mutations in each gene included in the targeted cfDNA sequencing of breast cancer patients. Each vertical line represents an individual patient sample, and the bar plot at the top indicates the number and distribution of mutations per sample (TMB, tumor mutation burden). Summarized clinical information for each sample is shown at the bottom. The bar plot on the right displays the number and distribution of mutations for each gene. Mutation types are color-coded as indicated in the legend. (**B**) Distribution of variants detected in coding regions and the top 10 most frequently mutated genes. (**C**) Distribution of variants detected in non-coding regions. SNP, single-nucleotide variant; INS, insertion; DEL, deletion.
