# Supplementary material for: Transcriptionally Informed Nucleosome Profiling of Circulating Cell-Free DNA Predicts Breast Cancer Recurrence
Source: Cancer Res Commun. 2026 Jun 15;6(6):1405–14. doi: 10.1158/2767-9764.CRC-26-0263 (PMC13266714; doi:10.1158/2767-9764.CRC-26-0263)
Supplement: Supplementary Figure S4 — Figure S4. Comparison of mutational signatures between primary and recurrent samples. [file crc-26-0263_supplementary_figure_s4_suppsf4.pdf]

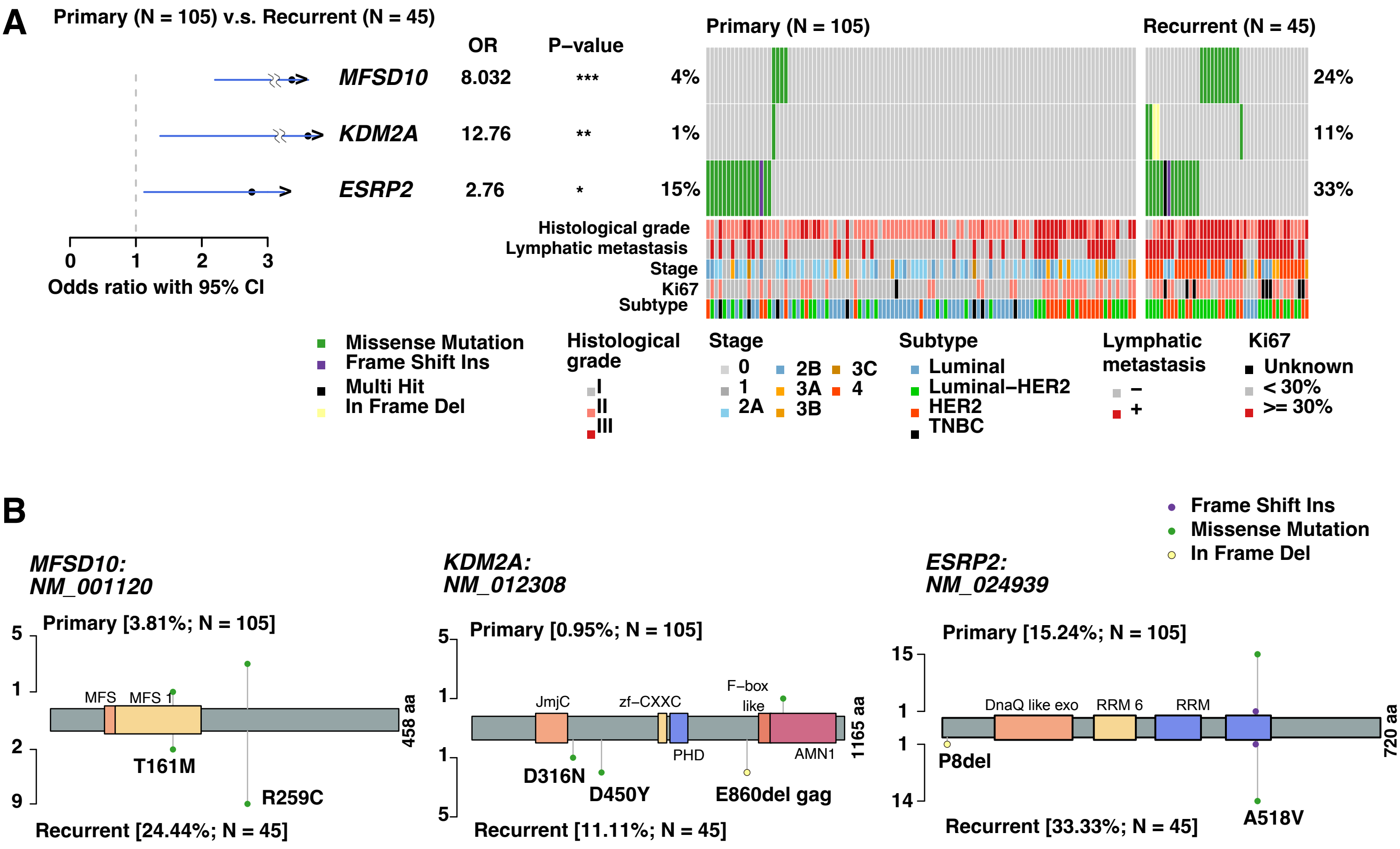


C

| gene | Mutation | dbSNP | Allele Frequency (gnomAD v4.0.0) |
|---|---|---|---|
| ***MFSD10*** | T161M | rs370161671 | 0.00009301 |
| | R259C | rs201057296 | 0.000455 |
| ***KDM2A*** | D316N | | |
| | D450Y | rs1194154891 | 0.000001591 |
| | E860del | rs766427768 | 0.000004108 |
| ***ESRP2*** | P8del | | |
| | A518V | rs3743738 | 0.002719 |

**Supplementary Figure S4 Watanabe et al.**

**Supplementary Figure S4. Comparison of mutational signatures between primary and recurrent samples.**

**(A)** Forest plot and co-oncoplot comparing genes differentially mutated between primary and recurrent samples. Mutation types and clinical information for each sample are indicated at the bottom of the panel. (**B)** Lollipop plots showing the positions of variants within each gene. Grey bars represent the full length of each protein, and colored boxes indicate functional domains. The height of each vertical line corresponds to the total number of variants identified in each amino acid position. (**C**) Table summarizing amino acid substitutions differentially enriched in recurrent samples.
