# Supplementary material for: Transcriptionally Informed Nucleosome Profiling of Circulating Cell-Free DNA Predicts Breast Cancer Recurrence
Source: Cancer Res Commun. 2026 Jun 15;6(6):1405–14. doi: 10.1158/2767-9764.CRC-26-0263 (PMC13266714; doi:10.1158/2767-9764.CRC-26-0263)
Supplement: Supplementary Figure S5 — Figure S5. Comparison of non-coding variant sites and their counts between primary and recurrent samples. [file crc-26-0263_supplementary_figure_s5_suppsf5.pdf]

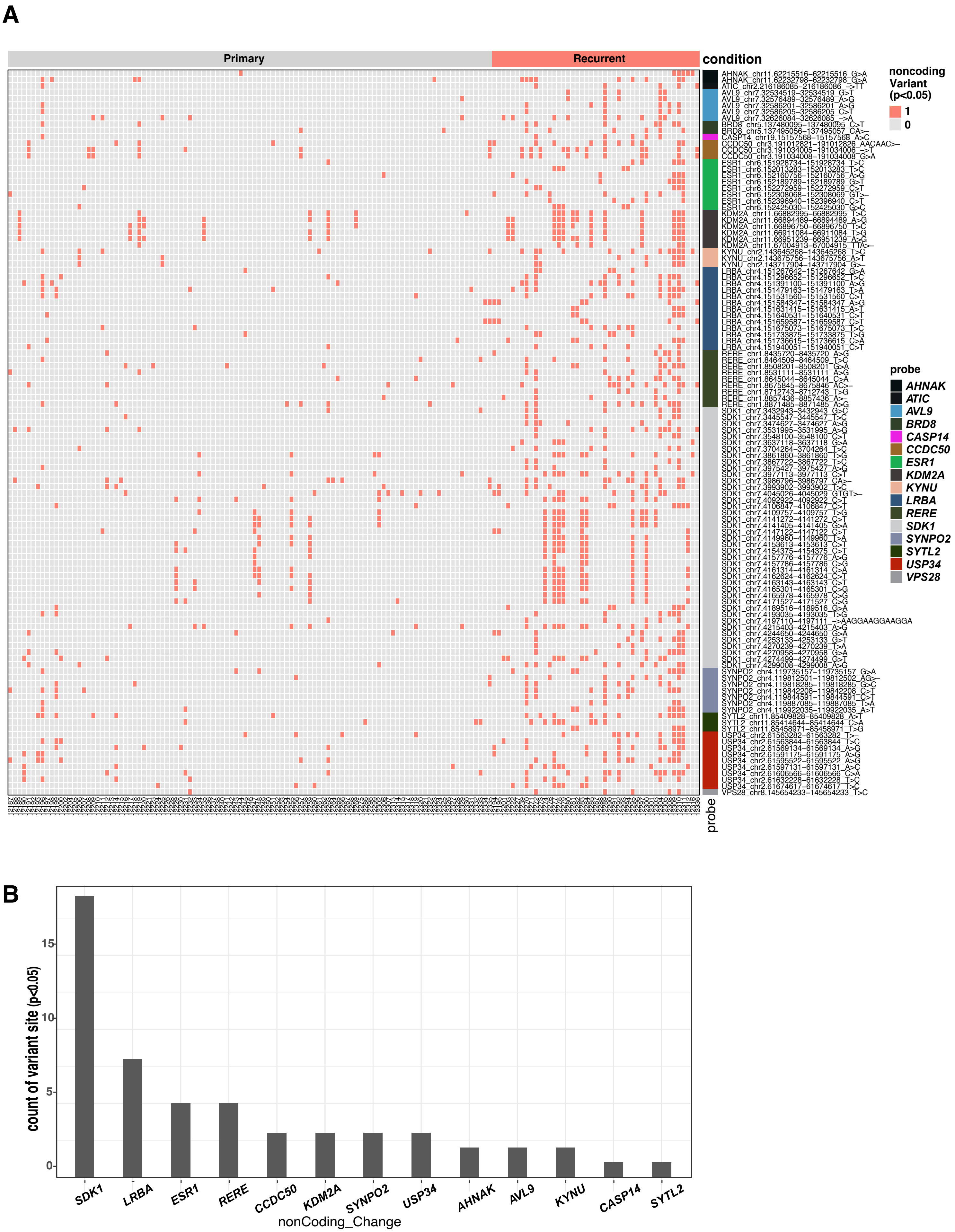

Supplementary Figure S5 Watanabe et al.

Supplementary Figure S5. Comparison of non-coding variant sites and their counts between primary and recurrent samples.

(A) Oncoplot depicting the location of variant in non-coding regions of each gene. (**B)** Bar plot showing the number of non-coding variant sites that were differentially increased in recurrent samples.
