# Supplementary material for: Transcriptionally Informed Nucleosome Profiling of Circulating Cell-Free DNA Predicts Breast Cancer Recurrence
Source: Cancer Res Commun. 2026 Jun 15;6(6):1405–14. doi: 10.1158/2767-9764.CRC-26-0263 (PMC13266714; doi:10.1158/2767-9764.CRC-26-0263)
Supplement: Supplementary Figure S6 — Figure S6. Oncoprint analysis of the 26 targeted loci in clinical breast cancer samples from the TCGA study. [file crc-26-0263_supplementary_figure_s6_suppsf6.pdf]

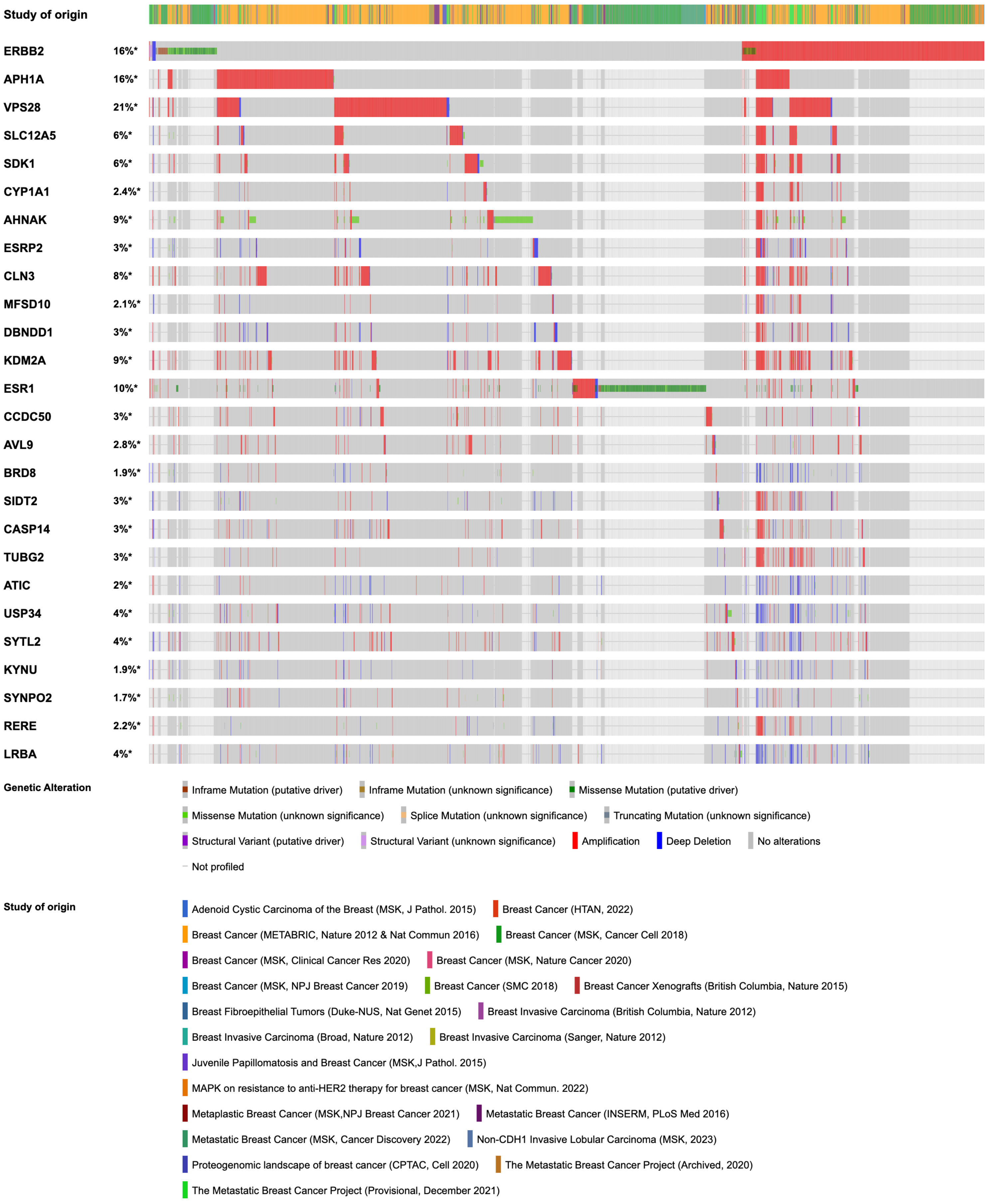

Supplementary Figure S6 **Watanabe et al.**

Supplementary Figure S6. Oncoprint analysis of the 26 targeted loci in clinical breast cancer samples from the TCGA study.
