# Supplementary material for: Transcriptionally Informed Nucleosome Profiling of Circulating Cell-Free DNA Predicts Breast Cancer Recurrence
Source: Cancer Res Commun. 2026 Jun 15;6(6):1405–14. doi: 10.1158/2767-9764.CRC-26-0263 (PMC13266714; doi:10.1158/2767-9764.CRC-26-0263)
Supplement: Supplementary Figure S7 — Figure S7. Quantification of cfDNA coverage across 26 genomic loci and nucleosome scores in the HER2-positive subset. [file crc-26-0263_supplementary_figure_s7_suppsf7.pdf]

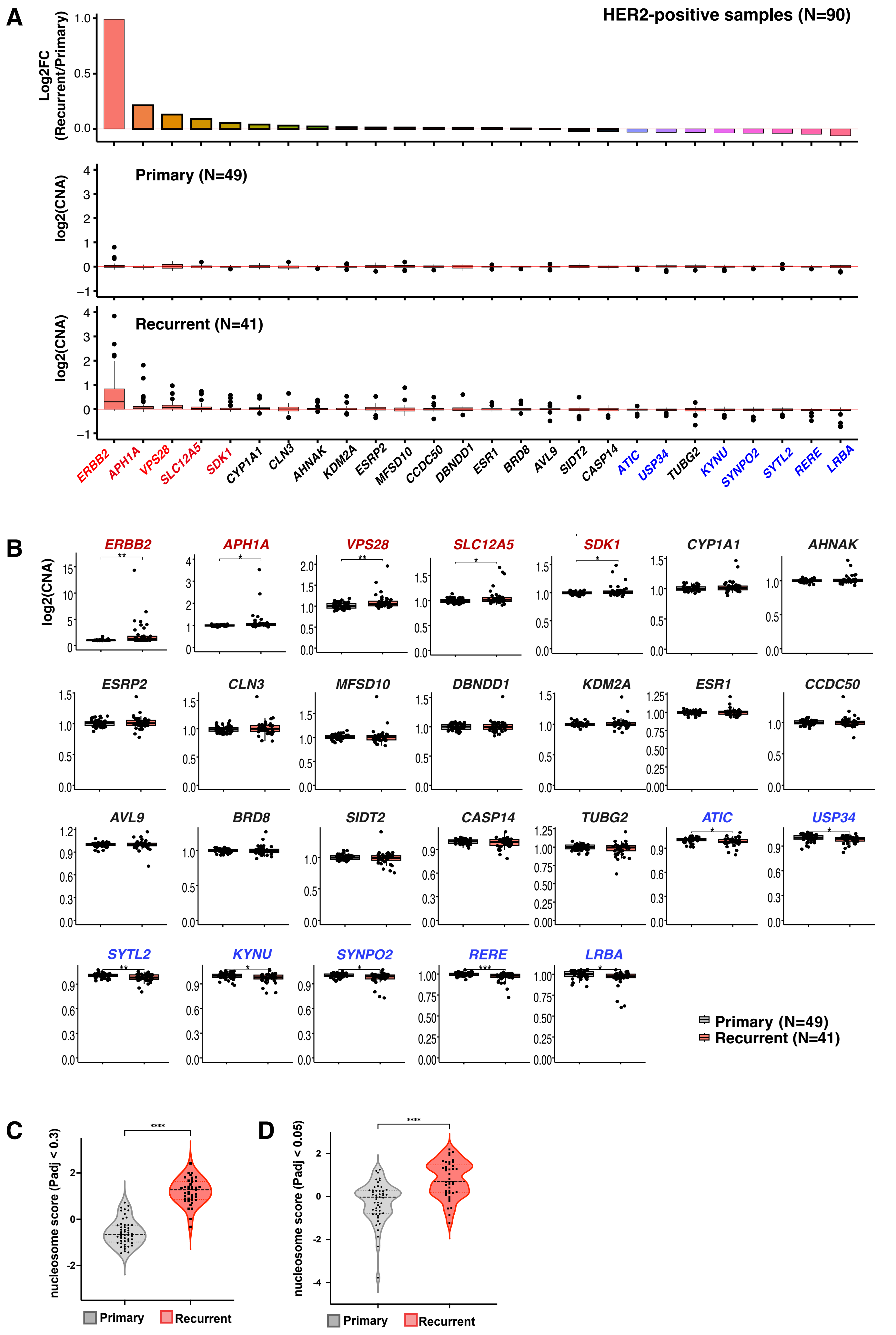

Supplementary Figure S7 Watanabe et al.

Supplementary Figure S7. Quantification of cfDNA coverage across 26 genomic loci and nucleosome scores in the HER2-positive subset.

(**A**) Quantification of cfDNA coverage across the 26 genomic loci. **(B)** Comparison of cfDNA coverage at targeted loci between primary and recurrent/metastatic samples. **(C**and **D)** Comparison of nucleosome scores between primary (N = 49) and recurrent (N = 41) samples. Nucleosome scores were calculated as z-scores of WPS across 19 genomic sites (Padj < 0.3) in (**C**), and across loci meeting a more stringent threshold (Padj < 0.05), including the *SYNPO2* locus and adjacent 10-nt windows upstream and downstream, in (**D**).
